# Associations of Common Variants in *HFE* and *TMPRSS6* Genes with Hepcidin-25 and Iron Status Parameters in Patients with End-Stage Renal Disease

**DOI:** 10.1155/2019/4864370

**Published:** 2019-03-07

**Authors:** Violeta Dopsaj, Aleksandra Topić, Miljan Savković, Neda Milinković, Ivana Novaković, Danica Ćujić, Sanja Simić-Ogrizović

**Affiliations:** ^1^Department of Medical Biochemistry, University of Belgrade - Faculty of Pharmacy, Belgrade 11221, Serbia; ^2^Center of Medical Biochemistry, Clinical Center of Serbia, Belgrade 11000, Serbia; ^3^Institute of Human Genetics, Medical Faculty, University of Belgrade, Belgrade 11010, Serbia; ^4^Department of Biology of Reproduction, Institute for the Application of Nuclear Energy (INEP), University of Belgrade, Belgrade 11080, Serbia; ^5^Clinic of Nephrology, Clinical Center of Serbia, Belgrade 11000, Serbia; ^6^Faculty of Medicine, University of Belgrade, Belgrade 11000, Serbia

## Abstract

**Background:**

Influence of *TMPRSS6* A736V and *HFE* (C282Y and H63D) polymorphisms on serum hepcidin-25 levels and iron status parameters in end-stage renal disease (ESRD) patients stratified according to gender has not been previously investigated. In addition, we aimed to evaluate the diagnostic accuracy of the parameters to separate iron-deficiency anemia (IDA) from anemia of chronic disease.

**Materials and Methods:**

Iron status parameters and genetic analysis were performed in 126 ESRD patients and in 31 IDA patients as the control group.

**Results:**

ESRD patients had significantly higher ferritin and hepcidin-25 (<0.001) relative to IDA patients. Cut-off values with the best diagnostic accuracy were found for hepcidin ≥9.32 ng/mL, ferritin ≥48.2 *μ*g/L, transferrin saturation ≥16.8%, and MCV ≥81 fL. Interaction between gender and *HFE* haplotypes for the hepcidin-25 and ferritin levels in ESRD patients (*p* = 0.005, partial eta squared = 0.09; *p* = 0.027, partial eta squared = 0.06, respectively) was found. Serum transferrin was influenced by the combined effect of gender and *TMPRSS6* A736V polymorphism in ESRD patients (*p* = 0.002, partial eta squared = 0.07).

**Conclusion:**

Our findings could contribute to the further investigation of mechanisms involved in the pathophysiology and important gender-related involvement of the *TMPRSS6* and *HFE* polymorphisms on anemia in ESRD patients.

## 1. Introduction

Anemia of chronic disease (ACD), called “anemia of inflammation”, is associated with infection, inflammation, malignancy, and in many conditions. It is well-known that inflammation in ACD could be associated with functional iron deficiency, characterized by normal or elevated body iron, locked in iron stores, and unavailable for erythropoiesis [1]. The classic sideropenic anemia is severe anemia with microcytic, hypochromic erythrocytes, with low serum iron, decreased transferrin saturation, and very low ferritin level. Also, the best way to diagnose anemia of chronic disease is to prove a mild or moderate decrease of hemoglobin with normocytic normochromic erythrocytes, low serum iron, and TIBC with normal transferrin saturation, as well as normal or elevated ferritin. To confirm the presence of coexisting iron deficiency (ID) in ACD, it is necessary to use more specific tests like sTfR and sTfR/index [2].

A molecular mechanism that explains the redistribution of iron during inflammation is connected with cytokine-stimulated overproduction of hepcidin. Recent studies have confirmed that hepcidin-25, a bioactive form of hepcidin, plays a central role in the regulation of iron metabolism and reduces the amount of iron in circulation [3–5]. Hepcidin synthesis is regulated at the transcriptional level by multiple stimuli [3]. Intracellular and extracellular iron concentrations increase hepcidin transcription, as does inflammation, whereas increased erythropoietic activity suppresses hepcidin production [4]. In turn, hepcidin regulates plasma iron concentrations by controlling ferroportin concentrations on iron-exporting cells including duodenal enterocytes, recycling spleen and liver macrophages, and hepatocytes [5–7].

Hepcidin deficiency is a result of autosomal recessive mutations in the hepcidin gene or genes encoding hepcidin regulators, the hemochromatosis gene (*HFE*, most commonly mutated in hereditary hemochromatosis in Caucasians populations), transferrin receptor 2 (TfR2), or hemojuvelin [8]. Consequently, the iron exporter ferroportin is overexpressed on the basolateral membranes of duodenal enterocytes which results in the development of systemic iron overload due to excessive iron absorption. The most frequent polymorphism of the *HFE* gene, located in the short arm of chromosome 6 (6p), is at position 845, where guanine (G) is replaced by adenine (A), resulting in the replacement of a cysteine by tyrosine at position 282 of the HFE protein sequence (C282Y), which leads to a nonfunctional protein [9]. The C282Y polymorphism prevents HFE protein from reaching the cell surface, thus preventing the interaction with hepcidin and TFRs [9]. The second most frequent *HFE* gene polymorphism consists of histidine to aspartic acid replacement at position 63 of the HFE protein sequence (H63D), due to a cytosine (C) to guanine (G) replacement at position 187. This polymorphism may disrupt iron homeostasis and cause iron accumulation when it cooccurs with the C282Y polymorphism [10].

The *TMPRSS6* gene, mapping to chromosome 22q12-q13, encodes for matriptase-2, a membrane-bound protease that decreases hepcidin transcription by cleaving hemojuvelin [11]. According to dbSNP genetic variations archive (NCBI), rs855791 identifies thymine (T) > C substitution in position 2207 (correspondent to a complementary A > G change), resulting the missense valine (V) to alanine (A) change in position 736 (p.V736A variant), nearby catalytic and binding sites of matriptase-2 [12]. Loss-of-function germline mutations of *TMPRSS6* cause iron-refractory iron-deficiency anemia. This is a rare type of anemia characterized by a lack of response to oral iron therapy but with partial response to parenteral iron administration [13]. Consequently, the change of the suppressive effect of TMPRSS6 protein on hepcidin production is characterized by extremely high hepcidin levels, whereas common rs855791 polymorphisms resulting in the p.A736V substitution is a significant determinant of iron status in healthy subjects and decrease hemoglobin concentration [12, 14].

Patients undergoing end-stage renal disease (ESRD) are commonly affected by anemia, which is related to erythropoietin (EPO) deficiency, blood losses, and chronic inflammation, which is the main difference compared to IDA [12]. Literature data suggest that renal anemia is normocytic and normochromic, often with coexisting iron deficiency [14]. ESRD patients are characterized by major alterations in iron metabolism including reduced iron availability for the erythroblasts and hyperferritinemia [15]. Upregulation of the serum levels of hepcidin has been proposed to explain the alterations of iron metabolism of ESRD patients and the resistance to anemia treatment [16, 17]. Epidemiological studies in the population of Serbia have shown that the prevalence of ESRD in adults is 0.07%, with commonly presented anemia of chronic disease [18].

This study aimed at evaluating serum hepcidin-25 concentration and iron status parameters in a population-based cohort of ESRD and IDA patients, as well as whether the polymorphism *TMPRSS6* A736V and *HFE* (C282Y and H63D) affect serum hepcidin-25 levels and iron status parameters in the analyzed patients.

## 2. Materials and Methods

### 2.1. Study Population

This cross-sectional study was conducted on 126 ESRD patients treated at the Clinical Center of Serbia, Clinic of Nephrology, between February 2016 and May 2017. According to the KDIGO guideline and glomerular filtration rate (GFR), all the analyzed patients were categorized as G5 stage of chronic kidney disease [19]. Patients were dialyzed with synthetic biocompatible membranes and bicarbonate dialysate three times per week. Patients were administered with *i.v.* synthetic erythropoietin (EPOetin alfa-Eprex), at a dose, aimed to maintain hemoglobin between 105 and 120 g/L. Iron was administered *i.v*. as Fe^3+^-hydroxide saccharose complex. Patients with IDA (*n* = 31) have been selected as control group, with hemoglobin concentration < 110 g/L, MCV < 80 fL, FRT < 20 *μ*g/L, and CRP < 10 mg/L [20]. The exclusion criteria for studied patients were known or suspected liver disease, immunosuppressive therapy, thalassemic trait, and patients with other known causes of anemia (e.g., inflammation and neoplasia). Moreover, we excluded from the study all patients aged <18 years, pregnant women, or patients with discordant antibodies. According to the report of the World Health Organization, the prevalence rate of sideropenic anemia is specifically higher among women in reproductive age which is why the IDA group was made up only of female patients [21]. Most frequently associated diseases with *TMPRSS6* and *HFE* studied alleles in ESRD population were hypertension and diabetes. Glomerulonephritis, polycystic disease, pyelonephritis, and other diseases (calculosis, sarcoidosis, gastritis, parathyroid disease, osteoporosis, and pulmonary and cardiac disease) were also present in lower degree. All patients gave informed consent to participate in the study, which was conducted according to the Helsinki Declaration and approved by the Ethics Committee of the Clinical Center of Serbia.

### 2.2. Biochemical Parameters

Venous blood samples for complete blood count, iron parameters, CRP, and genetic analysis were drawn via antecubital venipuncture and collected in the morning before hemodialysis, after one week after the last dose of *i.v.* iron, and three days after the last dose of EPO. Aliquots of the serum samples were stored at −80°C until the analysis.

Complete blood count was measured by flow cytometry on Coulter LH750 haematology analyzer (Beckman Coulter Diagnostics, USA). Urea, creatinine, iron, and unsaturated iron-binding capacity (UIBC) were determined by spectrophotometry and FRT, TRF, and CRP by a turbidimetric method on Olympus AU2700 analyzer (Beckman Coulter Diagnostics, Germany). We used the Modification of Diet in Renal Disease (MDRD) equation to estimate GFR [22]. Iron and UIBC values were used for TIBC calculation [23]. TSAT was calculated with the formula used iron and TIBC concentrations. Serum hepcidin-25 concentrations were determined using chemiluminescent direct ELISA technique (Corgenix, Inc., USA). We used each manufacturer's commercial control samples to check method specifications. Intra-assay CVs for all measured biochemical parameters were <5%.

### 2.3. Genetic Analysis

DNA was extracted from the blood samples with Na-citrate as anticoagulant, using commercial QiAamp DNA Blood Mini kit (Qiagen Inc., Germantown, Maryland, USA). For genotypization of *HFE* genotype (C282Y and H63D variants) and the *TMPRSS6* rs855791 polymorphism, (p.A736V variant), TaqMan predesigned SNP assays (Thermo Fisher Scientific Inc., Waltham, MA, USA) were performed on Applied Biosystems 7500 Real-Time PCR instrument, and acquired data were analyzed.

### 2.4. Statistical Analysis

Normality of continuous variables was assessed by the one-sample Kolmogorov–Smirnov test (K-S test). According to the normality of distribution, we applied the appropriate tests. Independent sample *T*-test, Mann–Whitney *U* test, one-way ANOVA, and univariate analysis of variance (two-way ANOVA) with the appropriate post hoc analysis (least significant difference test and Sidak's multiple comparison test) were used for the comparison of continuous variables among the groups. Chi-square test was used for the analysis of categorical variables. Deviations of genotypes' distributions from Hardy–Weinberg equilibrium were assessed by *χ*^2^-test for each cohort or Fisher's exact test (if cases were <5). Receiver operating characteristic (ROC) curve analysis was used for the evaluation of iron status parameters diagnostic characteristics and for the determination of the optimal cut-off values which differentiate between iron-deficiency anemia and anemia of chronic disease. Statistical analysis was performed using IBM SPSS Statistics version 20.0 (Chicago, IL). A post hoc statistical power analysis was conducted using the QUANTO software, version 1.2 for SNPs, *TMPRSS6* A736V variants, and *HFE* gene variants (C282Y and H63D variants) [24]. The haplotype analysis of two SNPs of *HFE* genes (H63D and C282Y) was done with haplotype analysis software [25]. In this study, a value of *p* < 0.05 was considered statistically significant.

## 3. Results

Two different pathogenic types of anemia have been examined from the aspects of iron status parameters and the SNPs of three genes (*TMPRSS6* A736V, *HFE* C282Y, and *HFE* H63D). The iron-deficiency anemia (IDA) was investigated in a group of 31 sideropenic patients and a group of patients with ACD that included 126 ESRD subjects.

Clinical characteristics of these two patient groups were presented in Table 1. The obtained very high levels of serum creatinine and urea, as well as very low estimated glomerular filtration rates (eGFR), are the expected characteristics of the dialyzed patients. The ESRD group of patients showed significantly higher levels of CRP compared to patients with sideropenic anemia (about two times), which is an indicator of chronic inflammation in ESRD patients. Both patient groups had clinically manifested anemia, with approximately the same hemoglobin levels. Differences in an iron status profile obtained in two patient groups corresponded with the pathophysiology difference of the specific anemia. Thus, in the ESRD group of patients with ACD, we found significantly increased serum iron, transferrin saturation, and MCV, as well as decreased TIBC, transferrin levels, and soluble transferrin receptor levels. Significantly increased levels of ferritin (about 32 times) and hepcidin-25 (about 18 times) were obtained in ESRD patients in comparison to patients with sideropenic anemia.

There was no difference in the frequency distribution of *TMPRSS6* A736V, *HFE* C282Y *HFE* H63D gene variants, and *HFE* haplotypes between two groups of patients (Table 2). Frequencies of *TMPRSS6* A736V genotypes and alleles in both patient groups were consistent with Hardy–Weinberg equilibrium. In both investigated patient groups, there were missing rare homozygote variants for the *HFE* H63D G/G and for the *HFE* C282Y A/A.

Assuming the prevalence of ESRD in Serbian adults of 0.07% (commonly with anemia of chronic disease), a post hoc power analysis showed that the recruited 126 patients with ESRD and 31 patients with sideropenic anemia (iron-deficient anemia) have power for identifying the associations (log-additive model of inheritance) 66% for *TMPRSS6* A736V, 27% for *HFE* H63D, and 17% for *HFE* C282Y gene variants.

**Figure 1:**
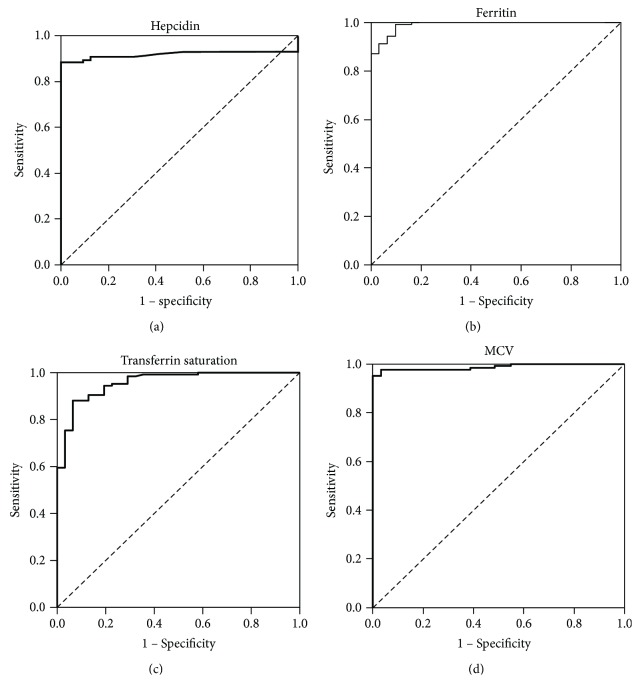
Receiver operating characteristic (ROC) curve analysis of diagnostic value iron-status biomarkers in distinguishing between IDA (sideropenic anemia) and ACD (ESRD patients): (a) hepcidin-25; (b) ferritin; (c) transferrin saturation; (d) MCV.

Receiver operating characteristic (ROC) curve analysis of diagnostic value of iron-status biomarkers in distinguishing between IDA (sideropenic anemia) and ACD (ESRD patients) was shown excellent diagnostic properties for hepcidin-25, ferritin, transferrin saturation, and MCV, with area under the curve (AOC) > 0.9 (Figure 1). Precisely, for hepcidin-25, AOC was 0.932 (St.error 0.021, *p* < 0.001); for ferritin, AOC was 0.991 (St.error 0.006, *p* < 0.001); for transferrin saturation, AOC was 0.958 (St.error 0.017, *p* < 0.001); and for MCV, AOC was 0.988 (St.error 0.007; *p* < 0.001). The cut-off values with the best diagnostic accuracy were obtained for hepcidin ≥9.32 ng/mL (specificity 100%, sensitivity 90%), for ferritin ≥48.2 *μ*g/L (specificity 100%, sensitivity 87%), for transferrin saturation ≥16.8% (specificity 84%, sensitivity 91%), and for MCV ≥81 fL (specificity 100%, sensitivity 90%).

ESRD patients with clinically manifested anemia showed gender-related differences in iron status, inflammation, and severity of renal disease (Table 3). In ESRD patients, males had higher serum iron levels, RBC and lower MCV, higher CRP levels, and higher eGFR.

There were no differences in the frequency distribution of gene variants of *TMPRSS6* A736V, *HFE* H63D, *HFE* C282Y, and *HFE* haplotypes between genders in the ESRD group of patients (Table 4).

The main results of this study were combined influence of gender and haplotypes of two *HFE* genes, as well as *TMPRSS6* A736V variants on the biomarkers of iron status.

**Figure 2:**
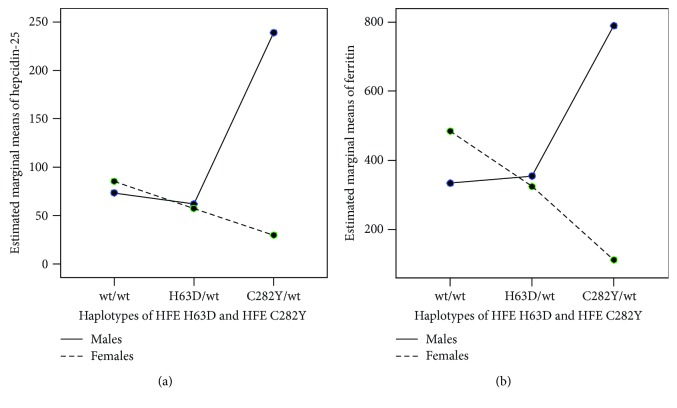
Gender-related effect of haplotypes of two *HFE* genes (*HFE* H63D and *HFE* C282Y) on the serum levels of (a) hepcidin-25 (ng/mL) and (b) ferritin (*μ*g/L) in patients with ACD (ESRD).

We found the statistically significant interaction between gender and haplotypes of two *HFE* genes (H63D/C282Y) on the serum levels of hepcidin-25 and ferritin in ESRD patients (*p* = 0.005, partial eta squared = 0.09; *p* = 0.027, partial eta squared = 0.06, respectively) (Figure 2). Using post hoc Sidak's multiple comparison test, we found that males with haplotype C282Y/wt has significantly increased levels of hepcidin-25 compared to males with haplotype H63D/wt (*p* = 0.002), males with haplotype wt/wt (*p* = 0.002), and females with the same haplotype C282Y/wt (*p* = 0.001) (Figure 2(a)). Additionally, gender-related differences in the serum ferritin levels showed that males with a haplotype C282Y/wt had higher ferritin level than females with the same haplotype (*p* = 0.029) (Figure 2(b)).

**Figure 3:**
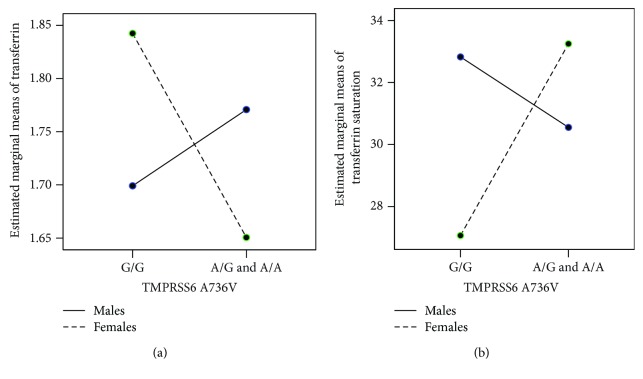
Gender-related combined effect of polymorphism of *TMPRSS6* A736V on the levels of (a) transferrin (g/L) and (b) transferrin saturation (%) in patients with ACD (ESRD).

The levels of serum transferrin were influenced by the combined effect of gender and *TMPRSS6* A736V gene variants in ESRD patients (*p* = 0.002, partial eta squared = 0.07) (Figure 3(a)). According to the *TMPRSS6* A736V gene variants, we analyzed a group of G/G homozygotes versus carriers of A allele (homozygotes A/A or heterozygotes A/G) according to the dominant model of inheritance. Females who carry a risk A allele have lower level of transferrin than G/G homozygote females (*p* = 0.002) and males regardless of the *TMPRSS6* gene variants (vs. G/G males, *p* = 0.018; vs. males with risk A allele, *p* = 0.048). The combined influence of gender and *TMPRSS6* A736V gene variants on transferrin saturation was shown in Figure 3(b). Although the influence was not significant and probability was very close to significance (*p* = 0.058, partial eta squared = 0.03), we found that transferrin saturation in females with risk A allele was higher than in females who were homozygotes G/G, but probability was close to significant (*p* = 0.063).

## 4. Discussion

Many literature data are dealing with the fact that hepcidin-25 is a significant iron status regulator and may serve as an important mediator in the pathogenesis of the anemia of chronic disease. In relation to this issue, there is a number of studies conducted in a population of patients with ESRD [14–17, 26, 27]. Direct measurement of the bioactive isoform of the hormone, hepcidin-25 in serum, is a reliable marker of iron homeostasis in this particular patient population because only in this form hepcidin is independent of glomerular filtration rate [28]. However, there is no final agreement whether hyperhepcidinemia is a constant and distinct feature of ESRD patients. Moreover, there is no consensus about the diagnostic characteristics for hepcidin-25 to distinguish IDA from ACD [17]. Probably the reason for this is that control groups in previous studies were not adequately selected. The inclusion of healthy participants in a control group on the basis of acute phase protein concentrations (CRP, FRT) and serum iron concentration is unreliable to exclude subclinical characteristics of anemia [17]. Regarding this, we have selected IDA patients with normal GFR and without inflammation (CRP <10 mg/L) as a control group, whereas ESRD patients were chosen on the basis of the present ACD.

We found excellent diagnostic properties for hepcidin-25, FRT, TSAT, and MCV for distinguishing IDA from ACD in ESRD patients. We presented the best diagnostic characteristic at 9.32 ng/mL for hepcidin-25 which is consistent with literature data [17, 26, 29]. Valenti et al. have stated that an adequate iron balance was likely present in most patients included in the second and third hepcidin quartiles (1.56–49.8 ng/mL) [17]. The concentration of hepcidin-25 which was obtained as the cut-off value in our study is in the lower quartile of the presented range and agrees with the result of Wagner et al. [27]. Additionally, FRT concentrations are mostly within recommended ranges for these patients and indicate the absence of inflammation. However, there is an extensive range for FRT cut-off values in iron deficiency, and it is difficult to exclude coexisting iron depletion, particularly in the presence of inflammation or underlying disease. In this study, we have obtained very high specificity and sensitivity at 48.2 *μ*g/L FRT concentrations, which is the expected value and in agreement with previously reported data [26, 30, 31]. We have obtained the best specificity and sensitivity for the cut-off value of 16.8% for TSAT. This value tells us that there is enough iron to meet the basic needs of erythropoietic tissue in our patients. Although recommended TSAT value of ≥20% principally refers to a healthy population, our lower result is expected because studied patients had a chronic renal impairment. Based on the RBC indices, anemia with MCV <80 fL was classified as microcytic and is characteristic for IDA, contrary to normochromic normocytic anemia which is a standard feature in ACD patients [32]. The obtained cut-off value for MCV, at 81 fL, with very high diagnostic properties in studied patients, agrees with literature data and presents an equally important parameter for distinguishing two types of anemia particularly in ESRD patients. Despite very high sensitivity and specificity, these parameters only jointly examined can predict iron status and could help in the administration of iron and erythropoiesis-stimulating agents in ESRD patients.

We found gender-related differences in iron status and inflammation in ESRD patients with clinically manifested anemia. Males with ESRD had an expected higher RBC and hemoglobin concentrations. Additionally, it is generally accepted that females had a higher prevalence of anemia than males, but that anemia in chronic kidney disease positively correlates with age in both genders [33]. This is because the population of ESRD patients included older persons, and the genetic effects on iron status from menstrual blood loss were minimized [34]. A possible reason that we did not find a difference in FRT concentrations in regard to gender is that female patients in our study were no longer in the reproductive period and had reduced blood loss. Tomschi et al. recently published that RBC indices were gender-related in elite athletes, i.e., persons with activated erythropoiesis [35]. Higher concentrations for MCV in the study of ESRD females could be a consequence of stimulated erythropoiesis as a response to therapy in our patients. In our study, we found lower CRP concentrations in female patients, which is in line with the literature data that point out the anti-inflammatory effect of estrogen [36, 37].

Recent genome-wide association studies show that very common single nucleotide polymorphisms in the *TMPRSS6* gene represent, together with *HFE* mutations, a significant determinant of the variation in iron status in the general population [38]. *TMPRSS6* A736V is common in the general population, with previous studies indicating a prevalence of 45% for the risk-associated T-allele [39]. However, it is confirmed that associations of common variants in *HFE* (C282Y and H63D) and *TMPRSS6* (A736V) genes with iron parameters are independent of serum hepcidin in a general population [40]. There are few literature data about frequency distribution and particular effect of these variants in ESRD patients. We found no difference in the frequency distribution of *TMPRSS6* A736V, *HFE* C282Y, and *HFE* H63D gene variants between IDA and ACD patients, which are in agreement with Pelusi et al. [15]. Patients that have been examined in this study lacked a rare homozygous variant *HFE* C282Y A/A and *HFE* H63D G/G. Several previous studies have reported that the prevalence of the homozygosity for *HFE* C282Y and *HFE* H63D mutations is very low in Southern and Eastern Europe [29, 41–43]. It is well-known that these variants are a common cause of hereditary hemochromatosis, and patients included in this study did not have this disorder. There are a number of studies trying to explain the connection of different common nonhematologic disorders with the frequencies of *TMPRSS6* and *HFE* gene variants [8, 16, 43–45]. Knowledge of genetic iron disorders can help to uncover other pathogenic or protective mechanisms in major human diseases.

To our knowledge, this is the first study that examines diagnostic characteristics for hepcidin-25 and iron status parameters in relation to *TMPRSS6* A736V, *HFE* C282Y, and *HFE* H63D polymorphism in ESRD patients. In this study, no differences were found in the frequency distribution of gene variants of *TMPRSS6* A736V, *HFE* H63D, *HFE* C282Y, and haplotypes of two *HFE* genes (H63D and C282Y) between genders. Some studies indicate that there is a gender-related difference in the distribution of *TMPRSS6* and *HFE* polymorphisms in some diseases [29, 44], but no studies have so far analyzed ESRD patients in this context. Moreover, we found higher serum levels of hepcidin-25 and FRT in males than in females in relation to *HFE* haplotypes. Literature data indicate that serum hepcidin concentrations in males are constant over the age and that they are higher compared to the concentrations in females, who have a tendency of higher hepcidin concentrations as they progress through menopause [6]. However, our results for the first time indicate a gender-related difference in hepcidin-25 levels in relation to the haplotypes of two *HFE* genes. Thus, male patients with haplotype C282Y/wt had the highest serum levels of hepcidin and ferritin. It seems that the presence of haplotype C282Y/wt in males lead to an increased hepcidin production, which in turn inhibits iron release from macrophages, with the consequently onset of anemia. Similarly, in a very large Danish Blood Donor Study, Sørensen et al. found that man heterozygous for either of the two *HFE* variants had higher ferritin levels than in wt type homozygous (the effect was higher for *HFE* C282Y), while no such difference was seen in women [46]. This observation could be clinically significant in the treatment of anemia in ESRD patients.

In addition, we found a significantly decreased level of serum transferrin with consequently increased transferrin saturation in ESRD females who are carriers of A allele (A/G and A/A) than females with wild-type homozygotes G/G in *TMPRSS6* gene. According to our best knowledge, our results for the first time showed that TMPRSS6 736Val variant is associated with low levels of transferrin and high transferrin saturation in ESRD females with anemia. Nai et al. [39] observed that homozygosity for TMPRSS6 736Val (genotype A/A according to SNP nomenclature) is associated with significant higher levels of hepcidin-25, lower transferrin saturation, and lower serum iron than in TMPRSS6 736Ala homozygotes (genotype G/G according to SNP nomenclature) in healthy individuals. These authors also showed that the G allele of TMPRSS6 rs855791 inhibits hepcidin more efficiently than the A allele. However, Galesloot et al. showed that the influence of TMPRSS6 rs855791 variants to ferritin, serum iron, transferrin saturation, and TIBC was not dependent on serum hepcidin in general population, adjusted for age and gender [47]. They found that transferrin saturation was decreased and TIBC was increased in individuals with *TMPRSS6* A/G and A/A variants in comparison to *TMPRSS6* G/G homozygotes, which corresponded with the findings in our study that were obtained in females with ESRD. Gender-related difference in transferrin levels and transferrin saturation associated with *TMPRSS6* rs855791 variants in patients with ESRD-related anemia is potentially clinical relevant, but the pathophysiological mechanism should be examined in extensive studies.

A limitation of this study is a relatively small sample size, which influenced the power for identifying the associations of investigated gene variants between patients with iron deficiency and anemia of chronic disease. So it is necessary to continue further research with a larger number of samples.

In conclusion, in the assessment of anemia in ESRD, it is essential to perceive not only traditional iron status parameters but also hepcidin-25, as a critical regulator of iron status. It remains to be clarified whether the mechanism by which hepcidin regulates iron metabolism is affected by the primary disorder in patients with ESRD. Our findings could contribute to the further investigation of mechanisms involved in the pathophysiology of anemia in ESRD patients. Additionally, our results highlight the important gender-related involvement of the *TMPRSS6* and *HFE* polymorphisms on anemia in ESRD patients and could help further research to investigate whether primary disorder itself modifies patient's response to anemia.

## Figures and Tables

**Table 1:** Characteristics in two patient groups with IDA (sideropenic anemia) and ACD (ESRD patients).

Characteristic	Sideropenic anemia (*n* = 31)	ESRD patients (*n* = 126)	*p*
Gender (females) (%)	100	44	<0.001
Age (years)	42 (33-47)	61 (46-66)	<0.001
Serum urea (mmol/L)	4.00 (3.40-4.90)	19.90 (17.02-23.22)	<0.001
Serum creatinine (*μ*mol/L)	65.00 (58.00-70.00)	766.00 (592.25-913.25)	<0.001
eGFR (mL/min/1.73m^2^)	>60	6.00 (5.00-7.00)	—
Hepcidin-25 (ng/mL)	3.00 (2.74-3.05)	53.98 (18.82-110.51)	<0.001
Hemoglobin (g/L)	103.00 (97.30-111.70)	104.95 (95.82-113.78)	0.776
Serum iron (*μ*mol/L)	6.00 (3.90-9.90)	11.9 (9.85-15.55)	<0.001
TIBC (*μ*mol/L)	69.50 (62.60-73.30)	40.60 (35.18-47.52)	<0.001
Transferrin (g/L)	3.18 (2.87-3.30)	1.73 (1.56-1.91)	<0.001
TS (%)	8.40 (5.30-14.60)	29.80 (23.40-37.25)	<0.001
sTfR (mg/L)	2.43 (1.94-3.51)	1.25 (0.93-1.64)	<0.001
Ferritin (*μ*g/L)	8.00 (8.00-8.50)	256.70 (81.35-593.15)	<0.001
CRP (mg/L)	1.20 (0.50-3.00)	2.70 (1.50-7.90)	<0.001
RBC (×10^12^/L)	4.23 (4.09-4.54)	3.41 (3.15-3.74)	<0.001
WBC (×10^9^/L)	6.75 (5.66-8.45)	6.44 (5.55-7.80)	0.586
MCV (fL)	75.88 (71.01-78.00)	93.88 (91.00-97.16)	<0.001
RDW (fL)	17.05 (15.70-18.06)	15.02 (13.99-16.35)	<0.001

Continuous variables were presented as median (interquartile range); ESRD: end-stage renal disease; eGFR: estimated glomerular filtration rate; TIBC: total iron-binding capacity; TS: transferrin saturation; sTfR: soluble transferrin receptor; CRP: C-reactive protein; RBC: red blood cell count; WBC: white blood cell count; MCV: mean corpuscular volume; RDW: red cell distribution width.

**Table 2:** Frequency distribution of *TMPRSS6* A736V, *HFE* C282Y, and *HFE* H63D gene variants (expressed as %) in two patient groups with IDA (sideropenic anemia) and ACD (ESRD patients).

Genotype/allele	Sideropenic anemia (*n* = 31)	ESRD patients (*n* = 126)	*p*
*TMPRSS6 A736V*			
A/A	22.6	18.3	0.860
A/G	45.2	47.6	
G/G	32.3	34.1	
A allele	55.0	42.0	0.689
H-W (*χ*^2^, *p*)	0.241 (0.623)	0.07 (0.796)	
*HFE H63D*			
C/C	87.1	78.6	0.285
C/G	12.9	21.4	
G/G	0	0	
G allele	6.4	10.7	0.313
H-W (*χ*^2^, *p*)	0.147 (0.928)	1.808 (0.405)	
*HFE C282Y*			
G/G	93.5	95.2	0.720
G/A	6.5	4.8	
A/A	0	0	
A allele	3.2	2.4	0.143
H-W (*χ*^2^, *p*)	0.035 (0.982)	0.076 (0.962)	
*HFE haplotypes*			
wt/wt	0.81	0.74	0.546
C282Y/wt	0.06	0.05	
H63D/wt	0.13	0.21	

**Table 3:** Gender-related characteristics in patients with ACD (ESRD).

Characteristic	Females (*n* = 54)	Males (*n* = 72)	*p*
Age (years)	58.50 (43.25-66.00)	62.00 (47.25-67.75)	0.165
Serum urea (mmol/L)	19.85 (16.27-23.80)	19.95 (17.90-23.08)	0.530
Serum creatinine (*μ*mol/L)	715.50 (518.50-852.7)	801.50 (661.50-979.25)	0.006
eGFR (mL/min/1.73 m^2^)	5.00 (4.00-7.00)	6.00 (5.00-7.75)	0.060
Hepcidin-25 (ng/mL)	61.13 (22.94-110.62)	47.37 (13.93-110.91)	0.358
Hemoglobin (g/L)	101.75 (93.25-108.90)	107.25 (98.02-117.57)	0.012
Serum iron (*μ*mol/L)	12.95 (10.30-15.70)	11.20 (9.02-15.35)	0.091
TIBC (*μ*mol/L)	38.80 (34.40-46.45)	42.40 (36.65-48.17)	0.132
Transferrin (g/L)	1.65 (1.50-1.95)	1.77 (1.57-1.91)	0.379
TS (%)	29.00 (21.45-38.35)	30.10 (24.47-37.25)	0.641
sTfR (mg/L)	1.28 (1.08-1.73)	1.18 (0.82-1.56)	0.106
Ferritin (*μ*g/L)	304.20 (99.95-616.95)	214.90 (77.75-548.73)	0.455
CRP (mg/L)	2.00 (1.35-4.67)	4.20 (2.00-10.15)	0.006
RBC (×10^12^/L)	3.38 (3.02-3.58)	3.49 (3.24-3.94)	0.018
WBC (×10^9^/L)	6.31 (5.40-7.21)	6.75 (5.62-8.20)	0.141
MCV (fL)	94.94 (92.51-97.83)	93.12 (90.01-96.28)	0.027
RDW (fL)	14.72 (13.84-16.54)	15.27 (14.04-16.26)	0.317

Continuous variables were presented as median (interquartile range); ESRD: end-stage renal disease; eGFR: estimated glomerular filtration rate; TIBC: total iron-binding capacity; TS: transferrin saturation; sTfR: soluble transferrin receptor; CRP: C-reactive protein; RBC: red blood cell count; WBC: white blood cell count; MCV: mean corpuscular volume; RDW: red cell distribution width.

**Table 4:** Gender-related frequency distribution of *TMPRSS6* A736V, *HFE* C282Y, and *HFE* H63D gene variants (%) in patients with ACD (ESRD patients).

Genotype/allele	Females (*n* = 54)	Males (*n* = 72)	*p*
*TMPRSS6 A736V*			
A/A	14.8	20.8	0.653
A/G	48.1	47.2	
G/G	37.0	31.9	
A allele	39.0	44.0	0.377
H-W (*χ*^2^, *p*)	0.009 (0.923)	0.138 (0.710)	
*TMPRSS6 A736V (dominant model of inheritance)*			
G/G	37.0	31.9	0.551
A/G and A/A	63.0	68.1	
*HFE* H63D			
C/C	72.2	83.3	0.133
C/G	27.8	16.7	
G/G	0	0	
G allele	13.9	8.3	0.158
H-W (*χ*^2^, *p*)	1.407 (0.495)	0.598 (0.742)	
*HFE C282Y*			
G/G	94.4	95.8	0.717
G/A	5.6	4.2	
A/A	0	0	
A allele	2.8	2.1	0.720
H-W (*χ*^2^, *p*)	0.033 (0.984)	0.044 (0.977)	
*HFE Haplotypes*			
wt/wt	66.7	79.2	0.279
C282Y/wt	5.6	4.2	
H63D/wt	27.8	16.7	

## Data Availability

The authors declare that the data supporting the findings of this study are available within the article or are available from the corresponding author upon reasonable request.

## References

[1] Weiss G., Goodnough L. T. (2005). Anemia of chronic disease. *New England Journal of Medicine*.

[2] Thomas C., Thomas L. (2005). Anemia of chronic disease: pathophysiology and laboratory diagnosis. *Laboratory Hematology*.

[3] Kautz L., Nemeth E. (2014). Molecular liaisons between erythropoiesis and iron metabolism. *Blood*.

[4] Ellingsen T. S., Lappegård J., Ueland T., Aukrust P., Brækkan S. K., Hansen J. B. (2018). Plasma hepcidin is associated with future risk of venous thromboembolism. *Blood Advances*.

[5] Ganz T., Nemeth E. (2012). Hepcidin and iron homeostasis. *Biochimica et Biophysica Acta (BBA) - Molecular Cell Research*.

[6] Galesloot T. E., Vermeulen S. H., Geurts-Moespot A. J. (2011). Serum hepcidin: reference ranges and biochemical correlates in the general population. *Blood*.

[7] Girelli D., Nemeth E., Swinkels D. W. (2016). Hepcidin in the diagnosis of iron disorders. *Blood*.

[8] Hollerer I., Bachmann A., Muckenthaler M. U. (2017). Pathophysiological consequences and benefits of *HFE* mutations: 20 years of research. *Haematologica*.

[9] Katsarou M.-S., Latsi R., Papasavva M. (2016). Population-based analysis of the frequency of HFE gene polymorphisms: correlation with the susceptibility to develop hereditary hemochromatosis. *Molecular Medicine Reports*.

[10] Genetics Home Reference. https://ghr.nlm.nih.gov/gene/HFE.

[11] De Falco L., Sanchez M., Silvestri L. (2013). Iron refractory iron deficiency anemia. *Haematologica*.

[12] https://www.snpedia.com/index.php/Rs855791

[13] Bârsan L., Stanciu A., Stancu S. (2015). Bone marrow iron distribution, hepcidin, and ferroportin expression in renal anemia. *Hematology*.

[14] Drüeke T. B., Locatelli F., Clyne N. (2006). Normalization of hemoglobin level in patients with chronic kidney disease and anemia. *New England Journal of Medicine*.

[15] Pelusi S., Girelli D., Rametta R. (2013). The A736V TMPRSS6 polymorphism influences hepcidin and iron metabolism in chronic hemodialysis patients: TMPRSS6 and hepcidin in hemodialysis. *BMC Nephrology*.

[16] Dion S. P., Béliveau F., Morency L. P., Désilets A., Najmanovich R., Leduc R. (2018). Functional diversity of TMPRSS6 isoforms and variants expressed in hepatocellular carcinoma cell lines. *Scientific Reports*.

[17] Valenti L., Messa P., Pelusi S., Campostrini N., Girelli D. (2014). Hepcidin levels in chronic hemodialysis patients: a critical evaluation. *Clinical Chemistry and Laboratory Medicine*.

[18] Djukanović L., Aksić-Miličević B., Antić M. (2012). Epidemiology of end-stage renal disease and hemodialysis treatment in Serbia at the turn of the millennium. *Hemodialysis International*.

[19] Ketteler M., Block G. A., Evenepoel P. (2017). Executive summary of the 2017 KDIGO chronic kidney disease-mineral and bone disorder (CKD-MBD) guideline update: what’s changed and why it matters. *Kidney International*.

[20] Mikhail A., Brown C., Wiliams J. A. (2017). Clinical practice guideline anemia of chronic kidney disease. *Renal Association Clinical Practice Guideline–Anemia of Chronic Kidney Disease*.

[21] WHO (2015). *The Global Prevalence of Anaemia in 2011*.

[22] Levey A. S., Bosch J. P., Lewis J. B., Greene T., Rogers N., Roth D. (1999). A more accurate method to estimate glomerular filtration rate from serum creatinine: a new prediction equation. *Annals of Internal Medicine*.

[23] National Committee for Clinical Laboratory Standards (1990). *Determination of serum iron and total iron binding capacity; proposed standard. NCCLS document H17- P*.

[24] Gauderman W., Morrison J. (2007). Quanto 1.2: a computer program for power and sample size calculations for genetic–epidemiology studies. http://hydra.Usc.Edu/gxe.

[25] Eliades N. G., Eliades D. G. (2009). *HAPLOTYPE ANALYSIS: software for analysis of haplotypes data. Distributed by the authors*.

[26] Ueda N., Takasawa K. (2017). Role of hepcidin-25 in chronic kidney disease: anemia and beyond. *Current Medicinal Chemistry*.

[27] Wagner M., Ashby D. R., Kurtz C. (2015). Hepcidin-25 in diabetic chronic kidney disease is predictive for mortality and progression to end stage renal disease. *PLoS One*.

[28] Peters H. P. E., Laarakkers C. M. M., Swinkels D. W., Wetzels J. F. M. (2010). Serum hepcidin-25 levels in patients with chronic kidney disease are independent of glomerular filtration rate. *Nephrology Dialysis Transplantation*.

[29] Chikwanda E., Daka V., Simakando M., Kowa S., Kaile T. (2018). Evaluation of hepcidin as a biomarker for the differential diagnosis of iron deficiency anaemia and anaemia of chronic disease. *Asian Journal of Medical Sciences*.

[30] Jackson H. A., Carter K., Darke C. (2001). *HFE* mutations, iron deficiency and overload in 10 500 blood donors. *British Journal of Haematology*.

[31] Knovich M. A., Storey J. A., Coffman L. G., Torti S. V., Torti F. M. (2009). Ferritin for the clinician. *Blood Reviews*.

[32] Ingale S. V., Ullewar M. P., Ingale V. C., Upadhye J. J. (2017). Evaluation of anaemia. *International Journal of Research in Medical Sciences*.

[33] Kassebaum N. J., Jasrasaria R., Naghavi M. (2014). A systematic analysis of global anemia burden from 1990 to 2010. *Blood*.

[34] Rushton D. H., Barth J. H. (2010). What is the evidence for gender differences in ferritin and haemoglobin?. *Critical Reviews in Oncology/Hematology*.

[35] Tomschi F., Bloch W., Grau M. (2018). Impact of type of sport, gender and age on red blood cell deformability of elite athletes. *International Journal of Sports Medicine*.

[36] Ahmed S. B., Ramesh S. (2016). Sex hormones in women with kidney disease. *Nephrology Dialysis Transplantation*.

[37] Stenvinkel P., Wanner C., Metzger T. (2002). Inflammation and outcome in end-stage renal failure: does female gender constitute a survival advantage?. *Kidney International*.

[38] Hanson E. H., Imperatore G., Burke W. (2001). *HFE* gene and hereditary hemochromatosis: a HuGE review. *American Journal of Epidemiology*.

[39] Nai A., Pagani A., Silvestri L. (2011). TMPRSS6 rs855791 modulates hepcidin transcription in vitro and serum hepcidin levels in normal individuals. *Blood*.

[40] Benyamin B., Ferreira M. A. R., Willemsen G. (2009). Common variants in *TMPRSS6* are associated with iron status and erythrocyte volume. *Nature Genetics*.

[41] Canavesi E., Alfieri C., Pelusi S., Valenti L. (2012). Hepcidin and HFE protein: iron metabolism as a target for the anemia of chronic kidney disease?. *World Journal of Nephrology*.

[42] Merryweather-Clarke A. T., Pointon J. J., Jouanolle A. M., Rochette J., Robson K. J. H. (2000). Geography of *HFE* C282Y and H63D mutations. *Genetic Testing*.

[43] He M., Workalemahu T., Manson J. E., Hu F. B., Qi L. (2012). Genetic determinants for body iron store and type 2 diabetes risk in US men and women. *PLoS One*.

[44] Camaschella C. (2013). Iron and hepcidin: a story of recycling and balance. *Hematology*.

[45] McLaren C. E., McLachlan S., Garner C. P. (2012). Associations between single nucleotide polymorphisms in iron-related genes and iron status in multiethnic populations. *PLoS One*.

[46] Sørensen E., Rigas A. S., Thørner L. W. (2016). Genetic factors influencing ferritin levels in 14, 126 blood donors: results from the Danish Blood Donor Study. *Transfusion*.

[47] Galesloot T. E., Geurts-Moespot A. J., den Heijer M. (2013). Associations of common variants in *HFE* and *TMPRSS6* with iron parameters are independent of serum hepcidin in a general population: a replication study. *Journal of Medical Genetics*.

